# Editorial: Assessing the consequences of antidepressant treatments

**DOI:** 10.3389/fpsyt.2023.1185678

**Published:** 2023-04-18

**Authors:** Markus Kosel, Chong Guang Ng, Norio Yasui-Furukori

**Affiliations:** ^1^Department of Adult Psychiatry, Hôpitaux Universitaires de Genève (HUG), Genève, Switzerland; ^2^Department of Psychological Medicine, Faculty of Medicine, University of Malaya, Kuala Lumpur, Malaysia; ^3^Dokkyo Medical University, Mibu, Tochigi, Japan

**Keywords:** major depressive disorder, drug therapy, psychedelic agents, lithium, menstrual bleeding, citalopram, emotion regulation

The pharmacological treatment of major depressive disorder during the last decades has essentially been based on antidepressants modulating monoaminergic neurotransmitter systems (serotonin, noradrenaline, and dopamine) by reuptake inhibition and the blockade of monoamine oxidases. Therapeutic and side effects of such medications have been widely assessed. However, since ketamine, a dissociative anesthetic targeting primarily the glutamatergic neurotransmitter system, has been increasingly investigated as a potential antidepressant following the pioneering work of Berman and later Zarate and their co-workers, the interest in unconventional approaches to treat depression has been revived (1–3). Indeed, the approval of intranasal esketamine to treat resistant depression, but also the widespread use of intravenous ketamine in the same indication and also acute suicidality is certainly one of the major developments in this field during the last years (4).

The first article in the Research Topic “*Assessing the consequences of antidepressant treatments*” by Raison et al. addresses the effects of the naturalistic psychedelic use on depression, anxiety, and wellbeing in a cross-sectional online survey in 2,500 adults conducted in the United States of America. To our knowledge, this is one of the largest studies of its kind. On average, study participants indicated 39 lifetime uses of psychedelics whereas the preferred substances were psylocibin (52%), LSD (30%), Ayahuasca, DMT, and ketamine. Based on the data collected including the use of several clinical scales, the authors conclude that psychedelic use is associated with significant improvements in depressive and anxious symptoms. Thirteen percent of participants recorded at least one harm linked to psychedelic use, such as substance misuse and impulsive behavior (criminal, auto-, or heteroagressive). These results strengthen the idea that the careful prescription of psychedelics could be beneficial to patients with depression. However, specific modalities of their use as potential antidepressant treatments or facilitators in psychotherapeutic settings and how to minimize adverse effects and the emergence of addictive behaviors needs to be carefully evaluated. Indeed, according to a recently published treatment guideline, only preliminary results with a low level of evidence are available assessing the efficacy of serotonergic psychedelics in major depression (3, 5).

Sklivanioti Greenfield et al. assessed the effect of a single dose of 10 mg of escitalopram, a widely used selective serotonin reuptake inhibiting antidepressant drug, on affective tonus, intensity of emotion and emotion regulation. Forty-six adults were recruited from a non-clinical population in Sweden. The International Affective Picture System was used to induce emotions (fear and disgust). A baseline assessment of all participants included an emotional reappraisal task. Behavioral data, electrodermal activity (EDA), and functional near-infrared spectroscopy (fNIRS) recordings were collected. Results were compared between groups 5 h after the administration of either placebo or 10 mg of escitalopram. The authors conclude that escitalopram reduces emotion intensity for both fear and disgust compared to placebo. However, the effect of cognitive regulation was more important than the effect of escitalopram. EDA and fNIRS results suggest differential mechanisms of emotion regulation of cognitive and pharmacological approaches. This study sheds light on the complex regulation of emotions using a selective serotonin reuptake inhibitor as a probe and links insights into neurophysiological processes to potential clinical approaches (6).

In the third article of the Research Topic, Zhuo et al. assessed the association of mainly a large number of antidepressants and mood stabilizers with the risk of increased menstrual bleeding, based on data retrospectively collected during a 24 months period by the physicians of participating adults in China. This is a very important study addressing an often-overlooked side effect of many antidepressant medications: impairment of hemostasis, especially during menstruation. Almost 2,000 participants, the majority with major depressive disorders were included. Heavy menstrual bleeding occurred in 33% of participants. It was most strongly associated with the prescription of venlafaxine, duloxetine, and mirtazapine and even higher when combined with valproate. As the authors state, these results, based on retrospective data need to be confirmed by a prospective study, including a control group.

The final article by Fujikawa et al. is a case report about a special treatment setting: administration of lithium using a nasal tube in a patient with bipolar disorder. Standard lithium medication was crushed, suspended and administered through the nasal tube. However, this procedure resulted in a critically lowered blood lithium concentration.

In this Research Topic, four articles address very different but important aspects of the pharmacological treatment of depression: new substance-based approaches (psychedelics), modulation of emotions by a serotonergic medication, an often-overlooked unwanted effect of antidepressants and mood stabilizers (increased menstrual bleeding) and practical aspects of mood stabilizing treatment when *per os* medication is not possible. Much needs to be done in order to address the overall efficacy of antidepressant and mood stabilizing treatment and to better address specific individual needs while minimizing side effects. The articles presented in this Research Topic reflect some aspects of the current state and challenges in the field.

## Author contributions

All authors listed have made a substantial, direct, and intellectual contribution to the work and approved it for publication.
